# Metal Content in Textile and (Nano)Textile Products

**DOI:** 10.3390/ijerph19020944

**Published:** 2022-01-15

**Authors:** Iria Rujido-Santos, Paloma Herbello-Hermelo, María Carmen Barciela-Alonso, Pilar Bermejo-Barrera, Antonio Moreda-Piñeiro

**Affiliations:** The Group of Trace Element, Spectroscopy, and Speciation (GETEE), Institute of Materials iMATUS, Department of Analytical Chemistry, Nutrition, and Bromatology, Faculty of Chemistry, Universidade de Santiago de Compostela, Avenida das Ciencias, s/n, 15782 Santiago de Compostela, Spain; iria.rujido@usc.es (I.R.-S.); paloma.herbello@usc.es (P.H.-H.); mcarmen.barciela@usc.es (M.C.B.-A.); pilar.bermejo@usc.es (P.B.-B.)

**Keywords:** textile, metal content, microwave-assisted acid digestion, ICP-MS, dyeing, bleaching, flame retardant, nanofinishing

## Abstract

Metals, metallic compounds, and, recently, metallic nanoparticles appear in textiles due to impurities from raw materials, contamination during the manufacturing process, and/or their deliberate addition. However, the presence of lead, cadmium, chromium (VI), arsenic, mercury, and dioctyltin in textile products is regulated in Europe (Regulation 1907/2006). Metal determination in fabrics was performed by inductively coupled plasma-mass spectrometry (ICP-MS) after microwave-assisted acid digestion. The ICP-MS procedure has been successfully validated; relative standard deviations were up to 3% and analytical recoveries were within the 90–107% range. The developed method was applied to several commercial textiles, and special attention has been focused on textiles with nanofinishing (fabrics prepared with metallic nanoparticles for providing certain functionalities). Arsenic content (in textile T4) and lead content (in subsamples T1-1, T1-2, and T3-3) were found to exceed the maximum limits established by the European Regulation 1907/2006. Although impregnation of yarns with mercury compounds is not allowed, mercury was quantified in fabrics T1-2, T5, and T6. Further speciation studies for determining hexavalent chromium species in sample T9 are necessary (hexavalent chromium is the only species of chromium regulated). Some textile products commercialised in Europe included in this study do not comply with European regulation 1907/2006.

## 1. Introduction

Textile products can be contaminated with metals during both production and storage. In addition, manufacturers add intentionally metals or metallic compounds to textile products to achieve or improve specific functionalities. As example, approximately 80% of leathers are chrome-tanned [1,2], while vanadium, chromium, barium, lead, copper, cobalt, and nickel are used in textile dyeing [3,4], and iron and manganese are used in bleaching processes [5,6]. In addition, antimony compounds have been demonstrated to enhance the flame retardant properties of halogen compounds and commonly flame-retardant compounds used in textiles preparation [7,8]. Similarly, titanium can be present in some textile products after the Zirpro process with hexafluorotitanate salts for achieving fire-resistant textiles [8,9]. Furthermore, antimony can be in poly-ethylene terephthalate (PET) fibres since antimony compounds are used as catalysts in PET manufacture [10]. 

Currently, the textile industry pays great attention to the possibilities of using metallic nanoparticles (NPs) to improve the manufacture of fibres and to obtain fabrics with new or improved properties [11]. A new category has, therefore, been added to textile finishing, called “nanofinishing” [12]. The use of titanium dioxide nanoparticles (TiO_2_NPs) is an example of nanofinishing to improve the existing textile manufacturing procedures, specifically the scouring and bleaching processes of fibres (nanoscouring and nanobleaching). The photocatalytic activity of TiO_2_NPs leads to a fast degradation (oxidation by the photogenerated oxidant radicals, such as ^•^OH, RO^•^, and RO_2_^•^ formed on the surface of TiO_2_NPs under irradiation of light) of natural colouring matter and pigments in fabrics [13,14].

New functionalities in textiles which incorporate metallic NPs include self-cleaning characteristics (ZnONPs and TiO_2_NPs) [15,16], hydrophobicity (SiO_2_NPs and ZnONPs) [11,17,18], antibacterial properties (AgNPs, CuONPs, ZnONPs, and TiO_2_NPs) [11,19,20], UV blocking activity (TiO_2_NPs, ZnONPs, CeO_2_NPs, and Al_2_O_3_NPs) [11,21], and electromagnetic wave shielding (Cu-, Ni-, Fe-, and Co-based NPs) [21], among others.

Information about the composition of fibres is mandatory (European Regulation 1007/2011) [22], and the content of several metals is restricted in textile products in Europe. Maximum legal concentration of lead, cadmium, chromium (VI), and arsenic (and their compounds) in fabrics is 1 µg g^−1^, except for leather articles coming into contact with skin where chromium (VI) content shall not exceed the level of 3 µg g^−1^. Dioctyltin (DOT) in textile products which are in direct contact with the skin can not exceed 1000 µg g^−1^. In addition, impregnation of yarns with mercury compounds is not allowed [23]. On the other hand, the European Commission (2009/567/EC) establishes the ecological criteria for the award of the Community Ecolabel for textile products [24].

Metals can be released from textile products and can permeate through the skin, reaching the bloodstream, and accumulate in several organs or tissues. Counter ion, bond type, valence, pH [25], and dermal health [26] affect the percutaneous absorption of metals. Moreover, recent studies reported possible penetration of metallic NPs through skin, which may pose a risk to human health [27,28]. Besides the potential risk of metals and metallic NPs for humans, the presence of metallic NPs in textiles could imply a risk to the environment since several studies have pointed out their release from fabrics during the laundry [29,30,31,32,33,34].

Laser ablation inductively coupled plasma mass spectrometry (LA-ICP-MS) and total reflection X-ray fluorescence spectrometry (TXRF) [35,36] allow for fabric analysis without sample decomposition. On the other hand, other methods including ICP-MS and inductively coupled plasma optical emission spectrometry (ICP-OES) [35,37,38,39,40,41] require the application of acid digestion processes for multi-element determinations. Nitric acid has been commonly used in the decomposition of fabrics by microwave-assisted acid digestion [35,37,38,39,40] and convective (heating block) digestion [41]. The reported methods demonstrated the ability to digest efficiently common textiles based on cotton, wool, silk, polyester, flax, and hemp [35,38,39,41] but not for new fabrics which contain metallic NPs. Thus, more drastic conditions for microwave-assisted acid digestion (sample pre-treatment) and ICP-MS (analytes determination) have been proposed as a reliable method for multi-element assessment in textile products including new fabrics such those based on metallic NPs.

## 2. Materials and Methods

### 2.1. Instrumentation

Samples and reagents were weighed using an analytical balance ML 204T (Mettler Toledo, Columbus, OH, USA). The microwave-assisted acid digestion was performed in 100 mL closed Teflon vessels using an ETHOS PLUS microwave lab-station (Milestone, Sorisole, Italy). The total metal content was assessed using a NexION^®^ 300X ICP-MS (Perkin Elmer, Waltham, MA, USA) equipped with a SeaFastSC2 DX autosampler (Elemental Scientific, Omaha, NB, USA). The nebulisation system consisted of a Meinhard^®^ nebuliser and a cyclonic spray chamber thermostated by a Peltier refrigerator.

### 2.2. Samples

Type and composition of the studied clothing are listed in Table 1. The table also shows the number of subsamples for some textiles, as some of them were visually different (made with different fibres).

The textile products studied were bought from online marketplaces except for sample T6 (men’s cycling culotte) and sample T9 (compression socks with silver ions for diabetic people) which were purchased from a local shop and a pharmacy, respectively. All studied garments were manufactured in Europe, except T1, T2, and T3 (textile products fabricated in Bangladesh by the same company).

Each garment subsample was cut in pieces (5 × 5 mm approximately) with ceramic scissors and stored in plastic bags at room temperature in the dark.

### 2.3. Reagents

18 MΩcm ultrapure water was collected from a Milli-Q^®^ water purification system (Millipore, Bedford, MA, USA). 69% Nitric acid (Hyperpur), 33% hydrogen peroxide, 37% hydrochloric acid, and 40% hydrofluoric acid were supplied by Panreac (Barcelona, Spain). NexIon Setup Solution (10 μg L^−1^ of U, Pb, Mg, Li, In, Fe, Ce, and Be) and Memory Test 1 Solution (1000 mg L^−1^ of Al, Ca, Fe, K, Mg, Na and 20 mg L^−1^ of Ag, As, Ba, Be, Cd, Co, Cr, Cu, Mn, Ni, Pb, Se, Tl, V, Zn) were purchased from Perkin Elmer. Standards of Ti, Sn, Ge, Rh, and In (1000 mg L^−1^ each) were also from Perkin Elmer. Standards of B, Li, Mo, and Sb (1000 mg L^−1^ each) were supplied by Merck (Darmstadt, Germany). Y and Hg (1000 mg L^−1^ each) were purchased from Panreac (Barcelona, Spain) and Scharlau (Barcelona, Spain), respectively.

### 2.4. Microwave-Assisted Acid Digestion

Clothing samples (0.2000 g) were placed into PTFE digestion vessels and 8 mL of 69% nitric acid, 0.5 mL of 33% hydrogen peroxide, 0.5 mL of 37% hydrochloric acid, and 1 mL of 40% hydrofluoric acid were added. Operating conditions of the microwave-assisted acid digestion procedure are detailed in Table 2. Digested samples and blanks were made up to 25 mL with ultrapure water. The samples were digested in triplicate and one blank was performed at least for each set of digestions.

### 2.5. Total Metal Determination by ICP-MS

Daily adjustment of ICP-MS parameters such as torch position, quadrupole voltages, and nebulisation flow was performed to maximise the sensitivity. This adjustment was carried out before the measurement of samples and using a solution of Be, Ce, Fe, In, Li, Mg, Pb, and U (1 µg L^−1^). Operating and acquisition parameters of ICP-MS, as well as monitored ions, are listed in Table 3. Due to the low sensitivity of arsenic, a longer dwell time (200 ms) than that used for the other elements (50 ms) was selected. Polyatomic interferences were minimised using helium as a collision gas (either 1.0 mL min^−1^ or 4.0 mL min^−1^ flow rate depending on the monitored ion, Table 3). A solution containing 10 µg L^−1^ of internal standards (Ge, Y, Rh, and In) in 1% nitric acid was mixed with standards and samples by a T-shaped plastic piece before their introduction into the nebuliser.

Digested textiles were diluted 10-fold with ultrapure water before analysis except for Zn determination in T1, T2, and T3 samples (and their respective subsamples), and for Ti assessment in all samples (200-fold dilution). Multi-elemental standard addition calibration was performed to avoid matrix effects. The standard addition calibration covered the range of 0–100 µg L^−1^, except for the quantification of aluminium and iron (range of 0–5000 µg L^−1^).

## 3. Results

### 3.1. Microwave-Assisted Acid Digestion

Since nitric acid has been found adequate to decompose textiles based on cotton, wool, polyester, flax, and hemp [35,38,39,41], a mixture of 69% nitric acid 33% hydrogen peroxide was firstly used for microwave-assisted acid digestion of (nano)textiles (Table 1). However, fine white particles were observed in all digests, which impairs precision of the ICP-MS measurements. Therefore, other mineral acids such as hydrochloric acid and hydrofluoric acid were tested, and clear acid digests were obtained when using small volumes of both acids (0.5 and 1.0 mL of hydrochloric acid and hydrofluoric, respectively). Finally, a mixture of nitric acid/hydrogen peroxide/hydrochloric acid/hydrofluoric acid (8:0.5:0.5:1, volume ratio) was selected, and the microwave operating conditions are listed in Table 2.

### 3.2. Limit of Detection, Precision, and Analytical Recovery Assays

The 3σ/m and 10σ/m criteria were applied to calculate the limit of detection (LOD) and the limit of quantification (LOQ), respectively. Using these criteria, σ is the standard deviation of eleven measurements of a blank (1% nitric acid) by ICP-MS, and m is the slope of the standard addition calibration. The LOD and LOQ of developed method referred to the textile sample mass are shown in Table 4. LOQs ranged between 0.00427 (beryllium) and 6.33 µg g^−1^ (titanium). The high LOQ value for titanium was attributed to the high dilution required (1:200) for its analysis.

The precision of ICP-MS determinations for each element was evaluated by the relative standard deviation (RSD) of eleven measurements of an acid digest. RSDs for metal concentrations were within the range of 1–3% (Table 4), showing good precision for ICP-MS quantification.

The accuracy of ICP-MS assessments was based on the analytical recovery approach at several concentration levels by spiking acid digests at the selected metal concentrations (Table 4). Each spiked digest was analysed eleven times and analytical recoveries ranging from 90 to 107% were obtained (Table 4), which implies accurate ICP-MS determinations.

### 3.3. Metal Content in the Analysed Textiles

Metal content in the studied textile products assessed by ICP-MS after a microwave-assisted acid digestion is shown in Table 5. 

## 4. Discussion

Despite the fact that the fabrics were manufactured in several European countries and some of them (T1, T2, and T3) in Bangladesh, most of the metal levels were found to be similar in all textile samples. The presence of some metals can be directly related to their use (or their compounds’ usage) during fabrics production and/or functionalisation. However, some metals such as beryllium, mercury, and cadmium were detected at trace levels in some samples even though they have not been reported to have a specific role in the textile industry. Beryllium (concentrations lower than 0.0300 µg g^−1^), mercury (concentrations between 0.0818 and 0.375 µg g^−1^), and cadmium (concentrations from 0.103 to 0.319 µg g^−1^) were found, and their presence in some garments could imply contamination during the manufacturing, storage and/or selling of textiles. Beryllium concentrations in the analysed samples were lower than those reported by Rezyć et al. [38] in samples processed in the Croatian textile industry.

The presence of other metals in textiles can be related to their use in some stages of the production. This is the case of lithium (concentrations within the range of 0.109–0.699 µg g^−1^) since lithium grease is one of the most widely used lubricants in industry worldwide, and the use of lithium grease in mechanical devices during the manufacturing of garments could contaminate textiles with this metal.

### 4.1. Metals Used in the Dyeing Process

The presence of metals in textiles can be directly related to the dyeing process. That is the case of vanadium, since vanadium compounds are used as catalysts in the production of aniline black dye (CI 50440) [42,43]. Vanadium was detected in most of samples, and the range of concentrations was 0.0160–0.256 µg g^−1^ where samples made of black fibres showed the highest vanadium content [T4 (0.123 ± 0.0167 µg g^−1^), T6 (0.256 ± 0.0294 µg g^−1^), and T9 (0.143 ± 0.0000797 µg g^−1^)]. Another example is chromium, because several chromium-based dyes, such as chrome complexes with salicylic acid-based dyes (Mordant Yellow 1 and Mordant Yellow 5) give rise to mainly yellow and orange colours. In addition, Mordant Red 7, Mordant Red 19, Mordant Black 3, Mordant Black 9, Mordant Black 11, and Mordant Blue 13 dyestuffs, obtained by chroming aryl azo compounds, are also used in the textile industry [3,44]. Chromium was, therefore, quantified in all samples, except T1-1, and chromium concentration was within the range of 0.156–1.03 µg g^−1^, except in textile coded as T9, which showed a very high chromium content (974 ± 6.01 µg g^−1^). Except for this sample (T9), similar concentrations were obtained by Menezes et al. [37] in textile samples produced in Brazil and China.

In addition, barium sulphate (whitening colourant) and barium pigments can be used in the textile industry. Most of the analysed samples were found to contain barium, such as textiles T2-3 (53.9 ± 4.82 µg g^−1^) and T13 (0.758 ± 0.117 µg g^−1^) that contained the highest and lowest concentrations, respectively. Similarly, lead white and lead chromate pigments are also used in the textile industry [45]. Lead concentrations varied between 0.165 and 1.39 µg g^−1^.

Other metals such as copper, cobalt, and nickel are also used during the dyeing process as mordants [3]. This is the case of cobalt and nickel chlorides, as well as copper (I, II) sulphates [46]. These elements were in the studied samples at the concentration ranges as follows: cobalt from 0.00561 to 3.21 µg g^−1^, nickel between 0.0383 and 0.341 µg g^−1^, and copper in the range of 0.361–41.1 µg g^−1^. The concentrations of these elements were lower than those previously reported in the literature [37,38,39]. Similarly, stannous chloride is also used as a mordant in dyeing procedures [46], and certain organotin species have been reported as biocides in textile products [47]. Sixteen studied samples were found to contain tin which ranged from 0.0556 to 0.255 µg g^−1^, except for textile T4 (5.34 ± 0.199 µg g^−1^) and textile T9 (1.26 ± 0.0630 µg g^−1^).

### 4.2. Metals Used in the Bleaching Process

Iron can be used to form metal-complex dyes [3,46,48] but it is also used as a catalyst in the bleaching process [5,6] (hydrophobic components removal guarantees an optimum further dyeing). All studied textile samples have been found to contain iron, and textile T4 was the one with the highest level (57.1 ± 3.67 µg g^−1^). Furthermore, the presence of manganese in textiles is also related to the bleaching stage since several manganese complexes are used as catalysts to enhance this process [6]. Regarding manganese, textile samples coded as T6 and T4 showed the highest concentrations (85.1 ± 3.76 and 25.0 ± 1.10 µg g^−1^, respectively). The concentration of manganese in the remaining samples was in the range of 0.171–7.20 µg g^−1^.

### 4.3. Metallic NPs in the Textile Industry

Titanium dioxide is broadly used as delustering agent in synthetic fibres to reduce the lustre and transparency of yarns [49]. Furthermore, hexafluorotitanate salts [8,9,50] and TiO_2_NPs [51,52] can be used to achieve anti-flammable textiles. Besides, TiO_2_NPs provide self-cleaning [16,53,54,55,56], ultraviolet protection [56,57], and antibacterial activity [56,57,58] to fabrics. In addition, titanium and titanium dioxide (bulk and nanoparticulate) are whitening colourants. In general, titanium concentrations in the studied textiles ranged between 156 (T1-2) and 6223 (T6) µg g^−1^.

Samples coded as T7 and T8 were purchased from the same manufacturer (e-market) who specified that TiO_2_NPs were added to these textile products (children’s T-shirt) to obtain protection against ultraviolet radiation (Table 1). It was found that titanium concentrations were 2590 ± 20 and 2949 ± 67 µg g^−1^ for T7 and T8, respectively.

The manufacturers of the remaining samples did not specify the addition of titanium, which could mean that titanium was used to enhance fire retardation or opacity of fibres. Moreover, titanium could be present in these samples since they contain totally or partially white fibres (bulk titanium dioxide and TiO_2_NPs provide brightness and opacifying characteristics). Samples T10, T11, T12, T13, and T14 (T14-1, T14-2, and T14-3) were all-white clothes. Sample T6 was the sample with highest concentration of titanium, and it was half white and half black (one colour in each side of the piece of garment). In addition, titanium was present in T1-2, T2-2, T2-3, T3-2, and T3-3 which were subsamples that contain white fibres mixed with coloured fibres.

Regarding zinc, textiles coded as T1, T2, and T3 were purchased from the same manufacturer who specified that these garments contained ZnONPs (Table 1) to provide UV protection [59]. Therefore, these textiles showed very high zinc concentrations, ranging from 280 to 873 µg g^−1^. Zinc content in the remaining textiles was slightly lower than those levels found in samples T1 to T3, varying between 0.668 and 5.68 µg g^−1^.

Finally, several manufacturers of the studied textiles have specified the presence of silver ions or AgNPs in the garments since silver compounds offer biocide properties to the textiles [60]. In accordance with textile compositions given by the manufacturers, silver ions were present in the samples T4, T5, T6, and T9, whereas AgNPs were added to textiles T10, T11, T12, T13, and T14 (T14-1, T14-2, and T14-3) (Table 1). Silver concentrations in the above-mentioned samples ranged between 0.436 (T11) and 31.2 µg g^−1^ (T5). In addition, another two samples (T1-1 and T2-1) were found to contain silver, although silver ions/NPs were not listed in the textile composition.

### 4.4. Other Uses of Metals in the Textile Industry

The presence of metals such as molybdenum and antimony is related to their use in flame retardant formulations [7,8,61]. Molybdenum was quantified in twelve samples at concentrations lower than 0.600 µg g^−1^. The levels of Mo in the samples were lower than those reported by Rezyć et al. [38] whose found Mo concentrations between 0.04 and 9.93 µg g^−1^ in textile samples. However, antimony was present at high concentrations (0.301 to 218 µg g^−1^). As previously commented, high antimony concentrations in polyester fibres can be due to its use in the fabrication of polyethylene terephthalate (the most common type of polyester used in garments) [10]. A direct relationship was found between the presence of antimony and the percentage of polyester in the samples, and samples with higher polyester percentages than 50% (Table 1) were those with the highest antimony levels (higher than 100 µg g^−1^, samples T2-2, T2-3, T3-2, T3-3, T5, T6, T11, and T13).

Arsenic compounds are effective insecticides and herbicides and the use of organoarsenicals is a common practice to control weeds in cotton fields and as defoliants of cotton plants [62]. Arsenic was, therefore, found in six cotton-based textile samples (T2-3, T3-2, T3-3, T4, T14-1, and T14-3) and in one non-cotton-based fabric (T11) (Table 1). The concentration of arsenic in the studied textiles varied between 0.0556 and 0.250 µg g^−1^, except for sample T4 (15.8 ± 0.920 µg g^−1^), where high arsenic levels could be associated with high cotton percentage (80%) in the garment.

## 5. Conclusions

A microwave-assisted acid digestion procedure using a mixture of nitric acid, hydrogen peroxide, hydrochloric acid, and hydrofluoric acid has found adequate for decomposing a vast variety of textiles and (nano)textiles. ICP-MS multi-element determinations were found to be accurate (analytical recoveries within the range of 90–107%) and precise (RSDs varied between 1 and 3%).

The content of cadmium, lead, chromium (VI), arsenic, mercury, and dioctyltin in fabrics is regulated in Europe (Regulation 1907/2006). The concentration of arsenic found in textile T4 (15.8 ± 0.920 µg g^−1^) exceeded the maximum legal concentration of arsenic in textile products manufactured in Europe (1 µg g^−1^). Subsamples T1-1, T1-2, and T3-3 showed concentrations of lead which were close to the maximum legal limit or over it, 1 µg g^−1^ (1.04 ± 0.0584, 1.20 ± 0.179, and 1.39 ± 0.0751 µg g^−1^, respectively). Impregnation of yarns with mercury compounds is not allowed, but mercury was found in fabrics T1-2 (0.375 ± 0.0462 µg g^−1^), T5 (0.0818 ± 0.00544 µg g^−1^), and T6 (0.157 ± 0.00597 µg g^−1^).

On the other hand, speciation studies are necessary in the analysis of chromium. Hexavalent chromium is the only species of chromium regulated in textile products, and its maximum allowable concentration is 1 µg g^−1^. Total concentrations of chromium in textiles T4 and T6 were just in the maximum legal limit of hexavalent chromium, whereas sample T9 was over it (974 ± 6.01 µg g^−1^).

Regarding metals such as silver, titanium, and zinc, which are mainly added as NPs (AgNPs, TiO_2_NPs, and ZnONPs) in functionalised (nano)textiles, high levels have been found in some garments which were reported to contain NPs by the manufacturers. Further studies would be welcome in order to characterise the particle size distribution as well as the release of these metallic NPs under conventional washing (laundry) conditions.

## Figures and Tables

**Table 1:** Type of item, number of subsamples, and composition of studied textiles.

Code	Subsamples	Type	Manufacturing	Composition
T1	T1-1; T1-2	Long sleeve shirt	Bangladesh	70% Viscose, 30% Cotton; with SOLARSHIELD ZnO^®^ (UPF Index: 40+)
T2	T2-1; T2-2; T2-3	Long sleeve shirt	Bangladesh	65% Polyester, 35% Cotton; with SOLARSHIELD ZnO^®^ (UPF Index: 40+)
T3	T3-1; T3-2; T3-3	Long sleeve shirt	Bangladesh	65% Polyester, 35% Cotton; with SOLARSHIELD ZnO^®^ (UPF Index: 40+)
T4	-- (a)	Socks	Unknown (Europe)	80% Cotton, 13% Polyamide, 5% Silver, 2% Elastane
T5	-- (a)	Men’s T-shirt	Spain	100% Polyester with silver ions
T6	-- (a)	Men’s cycling culotte	Romania	82% Polyester, 18% Elastane; product treated with silver chloride (antibacterial biocide)
T7	-- (a)	Children’s T-shirt	Germany	50% Cotton, 50% Cellulose (ModalSun) (UPF Index: 30)
T8	-- (a)	Children’s T-shirt	Germany	46% Cotton, 46% Cellulose (ModalSun), 8% Elasthane (UPF Index: 50+)
T9	-- (a)	Socks	Spain	66% Polyamide, 10% Polyamide with silver ions, 24% Elastane
T10	-- (a)	Headband	Czech Republic	60% Cotton, 32% Polyester (Nanosilver^®^), 8% Elastane (Lycra^®^)
T11	-- (a)	Women’s underwear	Czech Republic	52% Polyester (COOLMAX^®^), 48% Polyester (Nanosilver^®^)
T12	-- (a)	Men’s underwear	Czech Republic	60% Cotton, 32% Polyester (Nanosilver^®^), 8% Elastane (Lycra^®^)
T13	-- (a)	Men’s undershirt	Czech Republic	52% Polyester (COOLMAX^®^), 44% Polyester (Nanosilver^®^), 4% Elastane (Lycra^®^)
T14	T14-1; T14-2; T14-3	Socks	Czech Republic	49% BIO Cotton, 19% Polypropylene, 20% Polyamide, 10% Polyester (Nanosilver^®^), 2% Elastane (Lycra^®^)

(a) Only one subsample.

**Table 2:** Operating conditions of microwave-assisted acid digestion of textiles.

Time (min)	Temperature (°C)
0–2	Room temperature–150
2–7	150
7–9	150–170
9–19	170
19–20	170–200
20–40	200

**Table 3:** ICP-MS conditions for metal assessment in digested clothing samples.

**Operating Parameters**	
Radiofrequency power (W)	1600
Plasma gas flow (L min^−1^)	16
Auxiliary gas flow (L min^−1^)	1.2
Nebulisation gas flow (L min^−1^)	0.9–1.1
Collision cell gas	He
**Acquisition Parameters**	
Replicates	3
Sweeps/Reading	20
Dwell time per AMU (ms)	50 (200 for As)
Integration time (ms)	1000 (4000 for As)
**Monitored Ions (*m*/*z*)**	
1 mL min^−1^ He	^7^Li, ^9^Be, ^55^Mn, ^63^Cu, ^98^Mo, ^107^Ag, ^111^Cd, ^138^Ba, ^202^Hg, ^208^Pb
4 mL min^−1^ He	^49^Ti, ^51^V, ^53^Cr, ^57^Fe, ^59^Co, ^60^Ni, ^66^Zn, ^75^As, ^118^Sn, ^121^Sb
Internal standards	^74^Ge, ^89^Y, ^103^Rh, ^115^In

**Table 4:** Methodology validation.

	LOD_method_ (µg g^−1^)	LOQ_method_ (µg g^−1^)	(Concentration)_added_ (µg L^−1^)	Analytical Recovery (%)	RSD (%)
Li	0.00324	0.0108	0.25, 0.5, 1	90 ± 3	1
Be	0.00128	0.00427	0.25, 0.5, 1	103 ± 6	2
Ti	1.90	6.33	1, 5, 10	92 ± 6	1
V	0.00322	0.0107	0.25, 0.5, 1	97 ± 4	2
Cr	0.0176	0.0588	0.25, 0.5, 1	95 ± 6	3
Mn	0.00236	0.00788	0.5, 1, 5	91 ± 5	1
Fe	0.136	0.455	12.5, 25, 50	91 ± 6	1
Co	0.00151	0.00505	0.25, 0.5, 1	96 ± 4	1
Ni	0.0107	0.0356	0.25, 0.5, 1	97 ± 5	2
Cu	0.00977	0.0326	5, 10, 25	97 ± 2	1
Zn	0.138	0.459	1, 5, 10	96 ± 4	1
As	0.0143	0.0476	0.25, 0.5, 1	94 ± 6	2
Mo	0.00249	0.00829	0.25, 0.5, 1	99 ± 3	1
Ag	0.0119	0.0397	25, 50	106 ± 4	1
Cd	0.00383	0.0128	0.25, 0.5, 1	101 ± 3	1
Sn	0.0101	0.0335	1, 5, 10	104 ± 4	1
Sb	0.0134	0.0447	10, 25, 50	95 ± 3	3
Ba	0.00567	0.0189	1, 5, 10	101 ± 4	1
Hg	0.0240	0.0800	1, 5, 10	107 ± 6	2
Pb	0.00479	0.0160	0.25, 0.5, 1	99 ± 2	1

**Table 5:** Metal concentration (µg g^−1^) in the studied fabrics measured by ICP-MS.

**Sample Code**	**Li**		**Be**		**Ti**		**V**		**Cr**		**Mn**	
T1-1	0.699 ± 0.114		<LOQ		<LOD		0.0687 ± 0.00612		<LOQ		5.82 ± 0.143	
T1-2	<LOD		<LOQ		156 ± 11.8		0.0613 ± 0.00897		0.272 ± 0.0206		4.25 ± 0.106	
T2-1	<LOD		0.0112 ± 0.000754		<LOD		<LOD		0.189 ± 0.0193		4.00 ± 0.216	
T2-2	<LOD		<LOD		1020 ± 9.127		0.0859 ± 0.0136		0.156 ± 0.00830		1.56 ± 0.0612	
T2-3	<LOD		0.00789 ± 0.00128		1085 ± 16.01		0.0609 ± 0.00913		0.528 ± 0.000931		6.17 ± 0.634	
T3-1	<LOD		0.0108 ± 0.000574		<LOD		0.0491 ± 0.00500		0.369 ± 0.0600		7.20 ± 0.0225	
T3-2	<LOD		<LOD		1154 ± 57.01		0.0518 ± 0.00281		0.363 ± 0.0229		3.02 ± 0.0876	
T3-3	0.422 ± 0.0842		0.0237 ± 0.00400		1262 ± 24.85		0.0366 ± 0.00356		0.369 ± 0.0266		5.24 ± 0.300	
T4	<LOD		<LOD		567 ± 31.1		0.123 ± 0.0167		1.03 ± 0.00260		25.0 ± 1.10	
T5	<LOD		<LOD		1706 ± 5.244		0.0603 ± 0.00956		0.451 ± 0.0507		0.243 ± 0.0323	
T6	<LOD		0.0101 ± 0.000414		6223 ± 12.07		0.256 ± 0.0294		1.03 ± 0.0983		85.1 ± 3.76	
T7	<LOD		<LOD		2590 ± 20.28		<LOD		0.318 ± 0.0154		0.171 ± 0.0269	
T8	<LOD		<LOD		2949 ± 67.23		0.0525 ± 0.000177		0.359 ± 0.0165		0.187 ± 0.0112	
T9	<LOD		<LOQ		2946 ± 50.77		0.143 ± 0.0000797		974 ± 6.01		1.93 ± 0.0188	
T10	0.109 ± 0.0122		<LOD		464 ± 4.31		0.0325 ± 0.00263		0.995 ± 0.0323		0.726 ± 0.0336	
T11	0.186 ± 0.0255		<LOQ		1501 ± 24.28		0.0160 ± 0.00161		0.469 ± 0.0357		0.186 ± 0.0207	
T12	0.628 ± 0.102		0.0164 ± 0.000190		781 ± 11.6		0.0574 ± 0.0102		0.480 ± 0.0266		0.451 ± 0.0608	
T13	0.401 ± 0.0851		<LOD		2018 ± 57.69		0.0452 ± 0.00349		0.698 ± 0.0568		2.97 ± 0.0231	
T14-1	0.344 ± 0.0352		0.0235 ± 0.00459		887 ± 4.74		0.0471 ± 0.00227		0.358 ± 0.0233		3.97 ± 0.0658	
T14-2	0.263 ± 0.0618		0.0250 ± 0.000731		937 ± 10.1		0.0427 ± 0.00368		0.253 ± 0.0214		3.90 ± 0.00414	
T14-3	0.425 ± 0.0651		0.0202 ± 0.000320		426 ± 14.8		0.0486 ± 0.00737		0.206 ± 0.0104		0.823 ± 0.00837	
**Sample Code**	**Fe**	**Co**		**Ni**		**Cu**		**Zn**		**As**		**Mo**
T1-1	28.8 ± 1.49	0.0521 ± 0.00355		0.341 ± 0.0456		0.970 ± 0.00471		835 ± 27.3		<LOD		0.441 ± 0.0848
T1-2	30.7 ± 2.24	0.532 ± 0.00365		0.202 ± 0.0159		0.832 ± 0.0381		741 ± 12.7		<LOD		<LOD
T2-1	28.5 ± 0.0131	0.0143 ± 0.00143		0.265 ± 0.0147		0.818 ± 0.0470		458 ± 5.59		<LOQ		0.332 ± 0.0282
T2-2	27.7 ± 3.56	0.0572 ± 0.00801		0.142 ± 0.0224		2.75 ± 0.539		280 ± 0.591		<LOD		<LOD
T2-3	30.8 ± 3.91	0.496 ±0.0400		0.152 ± 0.0107		0.870 ± 0.106		518 ± 24.4		0.143 ± 0.0159		<LOD
T3-1	20.6 ± 1.38	0.0615 ± 0.00735		0.321 ± 0.0534		1.15 ± 0.127		873 ± 1.54		<LOD		0.306 ± 0.00300
T3-2	18.4 ± 0.0570	0.0825 ± 0.00596		<LOQ		15.6 ± 0.953		556 ± 5.06		0.0556 ± 0.0103		0.454 ± 0.0888
T3-3	31.7 ± 3.21	0.246 ± 0.0225		0.262 ± 0.0173		15.6 ± 0.619		713 ± 0.763		0.162 ± 0.00802		0.428 ± 0.0832
T4	57.1 ± 3.67	<LOD		0.334 ± 0.0454		41.1 ± 1.47		5.19 ± 0.368		15.8 ± 0.920		0.564 ± 0.0365
T5	15.3 ± 2.14	0.0583 ± 0.00152		<LOQ		0.373 ± 0.0375		1.01 ± 0.0974		<LOD		<LOQ
T6	27.8 ± 2.95	ND		0.262 ± 0.000818		0.747 ± 0.0609		5.68 ± 0.931		<LOD		<LOD
T7	5.20 ± 0.0757	0.00561 ±0.0000629		<LOQ		0.474 ± 0.00851		<LOD		<LOD		<LOD
T8	8.58 ± 0.23	<LOQ		0.166 ± 0.0246		2.46 ± 0.0794		ND		<LOD		0.257 ± 0.0516
T9	17.4 ± 2.36	0.127 ± 0.0129		0.179 ± 0.00262		1.61 ± 0.0418		5.45 ± 0.0912		<LOD		<LOD
T10	11.7 ± 0.0844	0.0846 ± 0.00339		<LOD		0.547 ± 0.0439		0.668 ± 0.00414		<LOD		0.223 ± 0.0205
T11	7.85 ± 1.12	3.21 ± 0.0895		0.0383 ± 0.000555		0.361 ± 0.0392		1.65 ± 0.0297		0.250 ± 0.00116		0.558 ± 0.0749
T12	25.6 ± 2.54	0.0275 ± 0.00144		0.151 ± 0.0252		0.415 ± 0.0256		<LOD		<LOD		0.0782 ± 0.0141
T13	18.7 ± 3.17	0.989 ± 0.0127		0.142 ± 0.00306		0.475 ± 0.0571		<LOD		<LOQ		0.266 ± 0.0224
T14-1	10.4 ± 0.371	0.0309 ± 0.00266		<LOD		0.559 ± 0.00213		1.73 ± 0.198		0.159 ± 0.0219		0.136 ± 0.0160
T14-2	14.2 ± 2.64	0.0328 ± 0.00407		<LOD		0.441 ± 0.0386		1.62 ± 0.0920		<LOD		<LOD
T14-3	11.9 ± 0.462	0.0233 ± 0.00183		<LOD		5.39 ± 0.146		0.819 ± 0.124		0.250 ± 0.0133		<LOD
**Sample Code**	**Ag**	**Cd**		**Sn**		**Sb**		**Ba**		**Hg**		**Pb**
T1-1	0.960 ± 0.154	0.301 ± 0.0120		<LOQ		0.649 ± 0.0181		3.20 ± 0.350		<LOD		1.04 ± 0.0584
T1-2	<LOD	0.276 ± 0.0130		<LOQ		22.0 ± 0.619		12.2 ± 0.498		0.375 ± 0.0462		1.20 ± 0.179
T2-1	0.959 ± 0.0906	0.189 ± 0.00272		<LOQ		0.463 ± 0.00564		5.90 ± 0.327		<LOQ		0.618 ± 0.0273
T2-2	<LOD	0.120 ± 0.00787		<LOQ		118 ± 1.61		1.28 ± 0.119		<LOD		0.552 ± 0.0490
T2-3	ND	0.244 ± 0.0260		0.162 ± 0.00119		133 ± 2.86		53.9 ± 4.82		<LOD		0.971 ± 0.0747
T3-1	<LOD	0.319 ± 0.00241		<LOQ		0.301 ± 0.0261		6.53 ± 0.334		<LOQ		0.888 ± 0.0415
T3-2	<LOD	0.186 ± 0.0104		0.0568 ± 0.00345		139 ± 4.86		1.92 ± 0.00587		<LOD		0.967 ± 0.0853
T3-3	<LOD	0.247 ± 0.00951		0.199 ± 0.0242		148 ± 1.70		6.45 ± 0.143		<LOD		1.39 ± 0.0751
T4	7.48 ± 0.235	<LOD		5.34 ± 0.199		8.12 ± 0.557		5.47 ± 0.698		<LOD		<LOD
T5	31.2 ± 1.64	0.103 ± 0.00833		0.105 ± 0.00907		109 ± 0.458		1.15 ± 0.149		0.0818 ± 0.00544		<LOD
T6	0.489 ± 0.0236	<LOQ		0.179 ± 0.0185		218 ± 15.9		ND		0.157 ± 0.00597		ND
T7	<LOD	<LOD		0.125 ± 0.000988		<LOD		ND		<LOD		<LOD
T8	<LOD	<LOD		0.255 ± 0.0482		<LOD		2.02 ± 0.357		<LOD		0.184 ± 0.0140
T9	5.83 ± 0.0668	<LOQ		1.26 ± 0.0630		16.1 ± 0.288		<LOD		<LOD		<LOD
T10	1.52 ± 0.0665	<LOQ		0.104 ± 0.00110		63.5 ± 0.961		<LOD		<LOD		<LOD
T11	0.436 ± 0.0260	<LOD		0.0556 ± 0.00472		143 ± 1.43		4.50 ± 0.511		<LOQ		0.657 ± 0.0237
T12	1.20 ± 0.171	<LOQ		0.0571 ± 0.00882		67.3 ± 2.80		ND		<LOQ		<LOD
T13	8.31 ± 0.137	<LOQ		0.0680 ± 0.00948		130 ± 0.935		0.758 ± 0.117		<LOD		<LOD
T14-1	6.40 ± 0.466	<LOD		0.136 ± 0.00799		33.7 ± 0.300		4.65 ± 0.766		<LOD		0.489 ± 0.0647
T14-2	6.02 ± 0.929	<LOD		0.133 ± 0.00236		34.3 ± 0.573		0.816 ± 0.142		<LOD		0.165 ± 0.0138
T14-3	3.47 ± 0.138	<LOQ		0.0760 ± 0.00782		15.3 ± 0.313		4.29 ± 0.568		<LOD		<LOD

<LOD: lower than limit of detection; <LOQ: lower than limit of quantification; ND: not determined.

## Data Availability

All the results obtained are shown in this article.
